# O.6.1-6 Process evaluation findings of the CHARMING intervention: exploring the involvement of community- and peer role models within a school-based physical activity intervention

**DOI:** 10.1093/eurpub/ckad133.265

**Published:** 2023-09-11

**Authors:** Kelly Morgan, Jordan Van Godwin, Bethan Pell, Rebecca Cannings-John, Rhiannon Tudor Edwards, Rachel Granger, Britt Hallingberg, Graham Moore, Esther van Sluijs, Holly Whiteley, Jemma Hawkins

**Affiliations:** Centre for Development, Evaluation, Complexity and Implementation in Public Health Improvement (DECIPHer), Cardiff University, UK; Centre for Development, Evaluation, Complexity and Implementation in Public Health Improvement (DECIPHer), Cardiff University, UK; Centre for Development, Evaluation, Complexity and Implementation in Public Health Improvement (DECIPHer), Cardiff University, UK; Centre for Trials Research, Cardiff University, UK; Centre for Health Economics and Medicines Evaluation, Bangor University, UK; Centre for Health Economics and Medicines Evaluation, Bangor University, UK; Cardiff School of Sport and Health Sciences, Cardiff Metropolitan University, UK; Centre for Development, Evaluation, Complexity and Implementation in Public Health Improvement (DECIPHer), Cardiff University, UK; MRC Epidemiology Unit, University of Cambridge School of Clinical Medicine, UK; vangodwinj1@cardiff.ac.uk; Centre for Health Economics and Medicines Evaluation, Bangor University, UK; Centre for Development, Evaluation, Complexity and Implementation in Public Health Improvement (DECIPHer), Cardiff University, UK

## Abstract

**Purpose:**

This study explored the involvement of community- and peer role models within the CHARMING (CHoosing Active Role Models to INspire Girls) intervention, aiming to increase and sustain physical activity among 9–10-year-old girls. CHARMING involves community role models delivering different 1-hour weekly taster physical activities with peer role models (e.g., older girls from secondary schools) supporting. Sessions take place after-school on the primary school premises over 6-weeks. The main research questions are i) Is it feasible and acceptable to recruit role models? and ii) What are the perceived barriers and facilitators to inclusion of peer role models within the intervention? This innovative research uses a theory-informed intervention which adopts a school-community partnership approach.

**Methods:**

A mixed methods process evaluation was embedded within a larger feasibility study, involving three secondary schools and four adjoining primary schools in South Wales, United Kingdom. One-to-one interviews were conducted with teachers (N = 10) across the seven schools and community role models (N = 10). Focus groups were conducted with 18 peer role models and 31 girls aged 9-10-years who participated in the intervention. Primary school teachers kept observation logs of each intervention session. A researcher completed observation logs of two random sessions per school. Qualitative data were analysed using thematic analysis with a combined deductive and inductive coding approach. Observation data were analysed using descriptive statistics. Data were triangulated and comparative analyses conducted across schools.

**Results:**

Twenty-three peer role models (aged 12-16-years) and 16 community role models participated in intervention delivery. Overall, the inclusion of both types of role models was shown as acceptable and feasible within the CHARMING intervention. Six themes were identified during analyses; reach and access, communication, logistics, existing systems, interpersonal relationships, and perceived impacts. Themes were intertwined across the barriers and facilitators of recruitment and implementation. Observation data highlighted intervention components which were not implemented consistently by role models. Areas for future improvement were highlighted.

**Conclusions:**

Findings can be used to optimise the CHARMING intervention and inform wider interventions or policies employing several role models across settings to promote physical activity among children.

**Funding Source:**

Health Care Research Wales Health Award (HRG-18-1494), DECIPHer (MR/KO232331/1).

